# Interstitial ectopic pregnancy, a rare clinical phenomenon diagnosed asymptomatically on routine work up in Tanzania: Case report

**DOI:** 10.1016/j.ijscr.2024.110688

**Published:** 2024-11-28

**Authors:** Murtaza Lamuwalla, Sajida Panjwani, Allyzain Ismail, Sunil Samji, Munawar Kaguta, Shweta Jaiswal

**Affiliations:** a Family Medicine Resident, The Aga Khan University, East Africa Medical College, Tanzania; b Department of Surgery, The Aga Khan Hospital, Dar-es-Salaam, Tanzania; c Department of Anaesthesia, The Aga Khan Hospital, Dar-es-Salaam, Tanzania; d Department of Obstetrics and Gynaecology, The Aga Khan Hospital, Dar-es-Salaam, Tanzania

**Keywords:** Interstitial ectopic pregnancy, Rare phenomenon, Tanzania, Case report

## Abstract

**Introduction:**

Interstitial ectopic pregnancy is a rare but life-threatening condition, accounting for 2.4 % of all ectopic pregnancies. Diagnosing it can be challenging, as the interstitial portion of the fallopian tube allows for delayed rupture due to its capacity to expand, often leading to significant haemorrhage. Early detection is critical to preventing severe complications.

**Case presentation:**

We report the case of a 30-year-old primigravida who presented for routine antenatal care at 12 weeks of gestation, asymptomatic with no identifiable risk factors. Routine ultrasound revealed a suspicious gestation near the left uterine horn, leading to a diagnosis of interstitial ectopic pregnancy via MRI. The patient underwent a laparotomy with successful wedge resection and salpingectomy. She recovered well without complications.

**Discussion:**

This case highlights the diagnostic challenges of interstitial ectopic pregnancy, which can remain asymptomatic longer than other types due to the anatomical features of the interstitial segment. Diagnostic imaging is vital for accurate diagnosis, especially in stable patients. Early intervention is key to avoiding catastrophic outcomes like uterine rupture and severe haemorrhage. Advances in imaging and surgical techniques have improved patient outcomes, but careful prenatal follow-up remains crucial for future pregnancies due to the increased risk of uterine rupture.

**Conclusion:**

Routine antenatal care and imaging play a pivotal role in the early detection of interstitial ectopic pregnancies. Prompt diagnosis and management can significantly reduce the risk of severe complications, emphasizing the importance of vigilant prenatal monitoring in reducing maternal morbidity and mortality.

## Highlights

• Rare finding of interstitial ectopic pregnancy
• Diagnosis on routine antenatal screening
• Asymptomatic until catastrophic
• Importance of components of antenatal screening

## 1 Introduction and importance

Ectopic pregnancy refers to an implantation of the embryo outside the uterine cavity and accounts for 1.2–1.4 % of all pregnancies [1]. Ectopic pregnancy can occur at different locations, `most commonly involving the ampullary (70 %), isthmic (12 %), fimbrial (11.1 %), ovarian (3.2 %), interstitial (2.4 %) and abdominal (1.3 %). Because only 2.4 % of ectopic pregnancies are interstitial in nature, it makes it one of the rarest locations for an ectopic pregnancy [2].

Clinical manifestations of ectopic pregnancies usually occur six to eight weeks after the last normal menstrual period, however may also occur later and presents most commonly with first trimester vaginal bleeding or abdominal pain. With regards to interstitial ectopic pregnancy it is more challenging to diagnose early as they may remain asymptomatic for a few more weeks as the interstitium can expand to a greater extent as compared to the rest of the tube [3]. Thus, they can be catastrophic when they rupture due to their size as well as high vascularity of the myometrium. As compared to other ectopic pregnancies it is more associated with severe haemorrhage with increased mortality with studies showing at least 25 % of such cases present in haemorrhagic shock [4].

The key risk factors associated with interstitial pregnancies are as those of other ectopic pregnancies which are, previous ectopic pregnancy, history of pelvic inflammatory disease or other genital infection, past tubal surgery and use of intrauterine devices. Due to low sensitivity and specificity of symptoms, it poses a diagnostic dilemma in modern medicine. Symptoms include pelvic, abdominal, or chest pain, vaginal bleeding, intra-abdominal bleeding, hypovolemic shock or uterine rupture. However most become symptomatic only after 12 gestational weeks at which time they may present with features of bleeding and shock [5].

It can be diagnosed through transvaginal ultrasound (US), Magnetic Resonance Imaging (MRI) or surgically. Using imaging it can be very difficult to distinguish interstitial pregnancy with intrauterine pregnancy or isthmus pregnancy due to its close proximity. Also, many present with rupture in shock necessitating emergent surgical exploration before imaging can take place [6]. Therefore, the importance of routine first trimester scans during initial visit to antenatal clinic so as to diagnosis such pregnancies before the present in a catastrophic situation. Despite national guidelines recommending at least one ultrasound scan within the first trimester studies show in sub Saharan Africa first-trimester contact rates are often below 25 % [7].

Management can either be expectant, medical using methotrexate or surgical by wedge resection or hysterectomy depending on the patient factors such as gestational age, size of the gestational sac, presence of cardiac activity and hemodynamic stability [8].

We present a case report of a rare interstitial ectopic pregnancy in a 30-year-old female who presented at our facility to book her Antenatal Clinic (ANC) at 16 weeks without any complains and upon routine workup was seen to have interstitial pregnancy. This paper has been reported in line with the SCARE 2023 criteria [9]. This article has been registered with the Research Registry.

## 2 Case presentation

A 30 years old Tanzanian female, primigravida at 12 weeks of gestational age presented to the ANC, having a confirmed pregnancy test done at a peripheral Hospital at 6 weeks gestational age. At time of her booking, she was otherwise healthy with no complaints nor identifiable risks. The patient reported no history of chronic illness, no gynaecological surgeries performed, no history of chronic medication use, no history of contraceptive method use, no history of sexual transmitted infection or pelvic inflammatory disease. Vital signs of the patient were normal at the time of presentation. Physical examination was unremarkable. No abdominal rigidity, involuntary guarding, or tenderness was found.

**Fig. 1:**
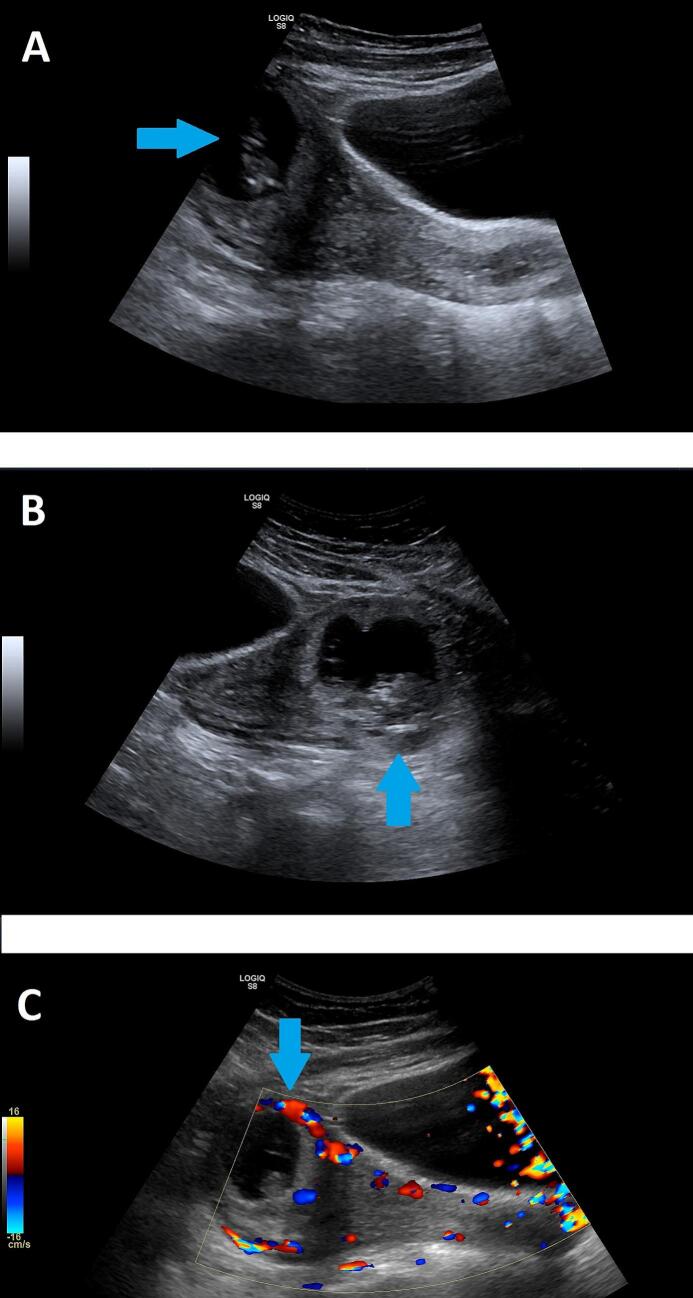
Transvaginal ultrasound of an interstitial ectopic pregnancy. A – Showing a gestation (Blue arrow) near the horn of the uterus. B – Showing a gestation (blue arrow) towards the left side with an empty endometrial cavity. C – Increased vascular Doppler flow (blue arrow) around the gestation.

**Fig. 2:**
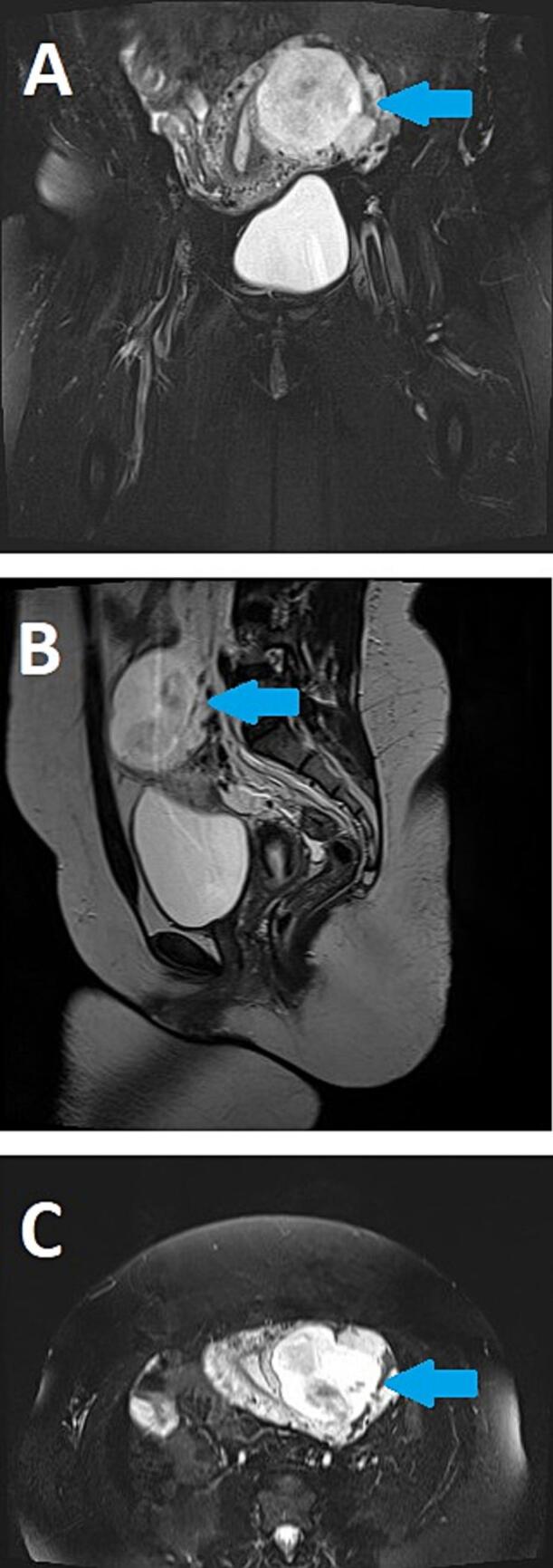
An MRI pelvis. A – Coronal view showing a heterogeneous signal intensity mass (Blue arrow) centered at the interstitial portion of the left fallopian tube immediately lateral to the left uterine angle. B – Sagittal view of a mass with a fetus within (blue arrow). C – Axial view of a mass on the left side with a fetus within (blue arrow).

Routine ANC investigations revealed no abnormality on blood work however the routine ultrasound revealed a gestation more towards the left side of the uterus with an empty endometrial cavity suspicious for an ectopic pregnancy verses a uterine abnormality (Fig. 1). Due to her hemodynamic stability, empty endometrium and inability to ascertain type of ectopic pregnancy or suspicion for a uterine abnormality an MRI was carried out for better anatomic description as it can delineate confounding findings seen on ultrasonography. The MRI revealed a centred gestation at the interstitial portion of the left fallopian tube immediately lateral to the left uterine angle with a foetus seen with a crown rump length of 5.8 cm corresponding to 12 weeks (Fig. 2). She was counselled on findings and due to risk of inevitable rupture with significant bleeding it was decided to undergo a laparotomy.

**Fig. 3:**
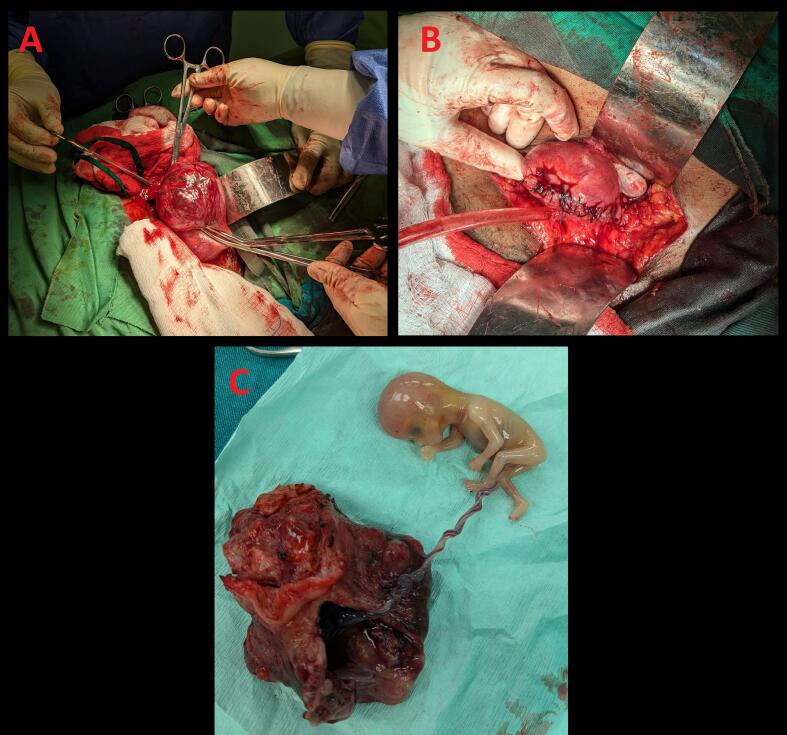
Intraoperative findings of an interstitial ectopic pregnancy. A – Unruptured interstitial ectopic pregnancy. B – Post wedge resection and left salpingectomy. C – Feotus within the gestational sac after wedge resection.

Under general anaesthesia, laparotomy was carried out and the diagnosis of an unruptured right interstitial pregnancy was confirmed (Fig. 3). Left wedge resection with left salpingectomy were performed and haemostasis was achieved. No complications were observed in the postoperative period and the patient was discharged from the hospital after 72 h of post-operative care. The patient was followed up as outpatient and no complications were reported. The patient was advised for close follow-up in her next pregnancy and to have a planned delivery due to risk of possible caesarean section due to risk of uterine rupture.

## 3 Discussion

Interstitial ectopic pregnancy, a rare but potentially life-threatening condition, accounts for 2–4 % of all ectopic pregnancies but contributes disproportionately to maternal morbidity and mortality [10]. It occurs when a fertilized ovum implants in the interstitial portion of the fallopian tube, the segment that passes through the muscular uterine wall. Due to its unique anatomical location, interstitial pregnancies pose diagnostic and therapeutic challenges, as illustrated by this case.

In this particular case, the patient presented asymptomatically for routine ANC work up and the diagnostic complexity of interstitial ectopic pregnancy lies in its initial asymptomatic period, which can be prolonged due to the more muscular and vascular nature of the interstitial portion of the fallopian tube. The delayed rupture associated with this condition leads to increased risk of massive haemorrhage and requires timely and accurate diagnosis. This underscores the importance of routine work up in all pregnant mothers at time of ANC visits. In Tanzania only 20 % attended their first ANC visit during the first trimester of pregnancy hence making the diagnosis during the asymptomatic stage even more difficult [11].

Ultrasonography proved instrumental in the diagnosis of interstitial pregnancy as it raised concerns due to the location of the gestation. Specific sonographic markers, such as the presence of an eccentrically located gestational sac near the uterine horn, the interstitial line sign (an echogenic line from the endometrium to the gestational sac) and incomplete myometrial coverage around the sac are signs pivotal in identifying the location of the pregnancy [6]. In clinically stable patients an MRI may be used to ascertain diagnosis and critical in distinguishing the interstitial pregnancy from other forms of ectopic pregnancy, particularly cornual and tubal.

An MRI can be used to help better define the location of the gestational sac with respect to the endometrium which can in some cases be difficult on sonography. On sonography findings of a gestational sac located in the lateral aspect of the uterine fundus surrounded by less than 5 mm of myometrium at the lateral aspect of the uterus should raise suspicion for an interstitial ectopic pregnancy [12]. Differences between interstitial pregnancy and angular pregnancy as well as identification of uterine anomalies are very subtle sometimes, and the sensibility of the conventional ultrasound is too low to distinguish them with expertise of the sonographer influencing findings [13]. Thus, an MRI can then be ordered for better anatomical delineation to confirm the diagnosis. However, an MRI is not easily performed due to the high cost, availability and long queuing time for appointments especially in resource limited settings. According to the World Health Organization's Global Atlas of Medical Devices, Tanzania has fewer than one MRI unit per million people [14]. This highlights a significant gap in diagnostic infrastructure hence the importance of having a high level of suspicion in patients with suspicious sonographic findings.

In terms of management, interstitial pregnancies pose unique challenges due to the high risk of uterine rupture and significant haemorrhage [15]. Historically, many cases of interstitial ectopic pregnancy resulted in hysterectomy or cornual resection, especially in cases where diagnosis was delayed, leading to rupture. However, advances in early diagnostic techniques and minimally invasive surgical approaches have improved patient outcomes. In this case, the patient's interstitial pregnancy was diagnosed at 12 weeks gestation prior to rupture, allowing for successful management without complications.

Following resolution of the interstitial pregnancy, it is important to counsel patients on the increased risk of recurrence in subsequent pregnancies and the potential for uterine rupture, particularly during labour [16]. Strict prenatal monitoring is recommended, and elective caesarean delivery may be advised to prevent uterine rupture. In this case, the patient was appropriately informed of the need for careful prenatal follow-up in future pregnancies.

## 4 Conclusion

In conclusion, this case underscores the importance of early detection and appropriate management of interstitial ectopic pregnancy to avoid catastrophic outcomes. While interstitial pregnancies are rare, their potential for severe complications requires clinicians to maintain a high index of suspicion. Routine workup at antenatal clinic has the potential to prevent catastrophic outcomes. Advances in imaging have significantly improved outcomes, but patient education and close follow-up remain crucial to mitigating the risks associated with this condition.

## Abbreviations

ANC: Antenatal clinic
MRI: Magnetic resonance imaging
US: Ultrasound

## Patient's perspective

I was very surprised that I had a problem in my pregnancy as I didn't have any symptoms but relieved to have been treated early. I understand the risks had it ruptured so am grateful everything worked out in the end.

## Consent

Written informed consent was obtained from the patient for publication of this case report and accompanying images. A copy of the written consent is available for review by the Editor-in-Chief of this journal on request.

## Provenance and peer review

Not commissioned, externally peer-reviewed.

## Ethical approval

Our institution does not require ethical approval for reporting individual cases or case series.

## Funding

This research did not receive any specific grant from funding agencies in the public, commercial, or not-for-profit sectors.

## Guarantor

Dr. Shweta Jaiswal.

## Research registration number

1. Name of the registry: RESEARCH REGISTRY
2. Unique identifying number or registration ID: researchregistry10786
3. Hyperlink to your specific registration: https://researchregistry.knack.com/research-registry#user-researchregistry/registerresearchdetails/6719627b8492b702c3639485/.

## CRediT authorship contribution statement

M.L.: Study conception, production of initial manuscript, collection of data, proofreading
S.P.: Revision of the manuscript, proofreading
A.I.: Revision of the manuscript, proofreading
S.S.: Revision of the manuscript, proofreading
M.K.: Production of initial manuscript, collection of data
S.J.: Study conception, production of initial manuscript, collection of data.

## Declaration of competing interest

None.
